# Socio-Cultural Influences on Situated Cognition in Nature

**DOI:** 10.3389/fpsyg.2019.00980

**Published:** 2019-05-03

**Authors:** Theresa Schilhab, Gertrud Lynge Esbensen

**Affiliations:** Future Technology, Culture and Learning, Danish School of Education, University of Aarhus, Copenhagen, Denmark

**Keywords:** situated cognition, attention restoration theory, learning, socio-cultural learning processes, nature, natural environments

## Introduction

To what extent are cognitive processes rooted in “simple” body-environment interactions, and the situation in which they take place? And to what extent does the body-environment interaction depend on socio-cultural processes?

Questions like these are pertinent to the field of environmental psychology, especially attention restoration theory (ART) (Kaplan and Kaplan, 1989; Kaplan, 1995). Here, concrete nature experiences are believed to incur certain attentional and cognitive states in the individual. Proponents of ART argue that self-regulation (Kaplan and Berman, 2010) and executive functioning involved in advanced cognitive operations like working memory, cognitive flexibility and attentional control (Diamond, 2013) gains from exposure to green environments. Recent meta analyses have pointed more specifically to the restoration of the system supporting so-called directed attention (Ohly et al., 2016; Stevenson et al., 2018).

The assumption is that the particular materiality of nature, e.g., the sounds, colors, and diversity (Fuller et al., 2007; Ratcliffe et al., 2013; Ossola and Niemelä, 2018), taps into our effortless stimulus-dependent attention at the expense of the directed (e.g., voluntary, sustained) attention we need in goal-directed tasks (e.g., Schilhab et al., 2018).

Accordingly, resting in nature leads to enhanced perceptual activity in a state of so-called soft fascination (Kaplan and Berman, 2010), reducing the time spent on problem-based cognition that involves the mentally fatiguing executive functions (Bratman et al., 2012) and inhibitory mechanisms to prevent external distractions (Diamond, 2013). This leaves time for the directed attention network to replenish (Stevenson et al., 2019). In this interpretation, natural stimuli work bottom-up by exteroceptive activation (Berman et al., 2008; Chun et al., 2011), irrespective of socio-cultural practices. As such, natural stimuli in the environment automatically trigger the particular cognitive state of soft fascination in an all or none fashion (e.g., Lee et al., 2015).

Based on ART, trips to the forest or park have become interventions to stimulate physical and mental health in children (McCurdy et al., 2010; Swank and Shin, 2015) and to relieve stress in adults (e.g., Corazon et al., 2011). Further, recent literature reviews agree that exposure to nature is generally beneficial to cognitive processing in a broad sense (Ohly et al., 2016; Stevenson et al., 2018).

However, the rather simple relationship between natural environments and cognitive states in ART raises questions about factors involved in body-environment interactions and situated cognition. What are the broader mechanisms governing green environments' ability to cause particular cognitive states? Does the materiality of nature work regardless of the meaning-making practices that occur in such environments? Although studies on individuals' favorite places for resting and self-regulation (e.g., Korpela et al., 2001), as well as studies on connectedness to nature, show that intersubjective variations exist (Mayer et al., 2009; Capaldi et al., 2014), explanations in environmental psychology and ART seldom include social or cultural modifiers of the nature-induced cognitive state (e.g., Auburn and Barnes, 2006). The extent to which we *learn* in childhood to categorize particular environments as “feasible” favorite places and as aids in self-regulation, or how to identify and appreciate “nature connectedness,” seems under-researched (however, see Adevi and Grahn, 2012).

Here, we point to and clarify a selection of possible sources that might influence situated cognition and thus explain deviations in, for instance, private preferences. Hence, we focus on possible areas of learning that may influence the “simple” body-environment interaction while resting in nature. The aim is to identify socio-cultural components and sources that are likely to moderate not only the relation of the natural environment with cognitive states in ART but situated cognition in general. Thus, we suggest that claims about the rooting of cognitive processes in bodily interaction with the environment would benefit from a consideration of the involvement of socio-cultural processes, similar to those we claim are pertinent to nature-induced cognitive states in ART.

## Levels of Socio-Cultural Influences

Following ART, when conditions are favorable, green environments elicit particular cognitive states in the individual^1^.

Apparently, this effect occurs automatically and with necessity, which paves the way for evolutionary inspired suggestions that rate green environments as more adaptive than urban and human-made settings (for a critique, see Joye and Van den Berg, 2011). Largely, the contention is that nature-induced cognitive effects depend on our prehistoric adaptation for bonding with and inhabiting green environments (e.g., Joye and De Block, 2011; Beery et al., 2015).

However, socio-cultural factors like meaning-making in situated social practices (Lave and Wenger, 1991), cultural learning processes in situated practices (Hasse, 2012, 2016), and the continuous forming of self-understanding in the individual, including motivations and emotions in relation to the surrounding social spheres (Holland et al., 1998), may modify the environmental impact on cognitive states. The presence of such socio-cultural factors questions any unconditional bottom-up causality in cognition. We therefore conjecture that socio-cultural processes co-determine the cognitive processes when perceiving a green environment, as suggested by (Lentini and Decortis, 2010, see also Nova, 2005; Clark and Uzzell, 2006)(p. 408):

in terms of *people's experience, sense of place refers to the fact that people apprehend physical space not only through the perception of its spatial characteristics, but also through the awareness of the social cues related to it*.

Overall, the socio-cultural approaches question the validity of claims about the impact of green environments on cognition in so far as these ignore the implicit or explicit connotations of green environments learned by the individual.

In the following, as a heuristic tool in order to exemplify, we divide the socio-cultural influences by how the individual learns about them^2^. For the sake of clarity, we distinguish between socialization through joint activities and talk when acting together in the moment, activities that often take the form of discursive and embodied learning (e.g., Auburn and Barnes, 2006), and socialization through socio-cultural imaginaries that seem more explicitly construed. Imaginaries can be viewed as “collectively held, institutionally stabilized, and publicly performed visions of desirable futures, animated by shared understandings of forms of social life and social order” (Jasanoff, 2015, p. 4). However, both kinds of socio-cultural processes are likely to influence cognitive processes simultaneously.

## Examples of Socio-Cultural Learning

Simply put, a child's very first bodily exposure to a green environment entails a concomitant exposure to the attitudes held by parents and caregivers toward this particular environment (e.g., Schilhab, 2015, 2018). The attitudes appear in the discourse surrounding the experience of the green environment, what is articulated and explicitly pointed to, and in the practices on the spot (for a neural description of the cognitive processes, see Schilhab, 2011, 2015a, 2017a).

According to the Russian psychologist Vygotsky, cultural development occurs initially on a social level (interpsychological) and only afterwards on an individual level (intrapsychological) (Vygotsky and Cole, 1978, p. 57).

If, say, the hooting of an owl is consciously noted by caregivers, then the presence of owls and their significance to the experience of nature is also emphasized, and the owl as phenomenon is attributed value (Tylén et al., 2010). This may explain why the presence of certain birds such as magpies and crows is negatively correlated with a subject's sense of recreation in green environments, although bird song is generally valued (Cox and Gaston, 2015; Gunnarsson et al., 2017).

In that sense, any momentary interaction with green environments involves both the processing of the materiality (e.g., the sight, sounds, smells, tactility, the kinaesthetic, and interoceptive responses) and the processing of the social interpretations (Barrett, 2009). Hence, families that use walks in green areas for leisure and pleasure will often socialize younger members into this particular green area mind-set. In such cases, the experience of a relaxed atmosphere and the attentive presence of parents become associated with spacious green stretches, experiences of freedom, bird song, and the smell of pine or blooming flowers, for example. Similarly, avid bird watchers or botanically skilled adults emphasize particular occurrences and events in concrete ways. These ways may also include certain technologies such as binoculars, cameras, taxonomic encyclopedias, or smartphone supported apps, while physically or meditatively minded adults corroborate either the physiological presence or the tranquility of the natural environment in sync with their particular perception (for preferences for particular sites, see Schebella et al., 2017).

The social glossing over of how to perceive and embody green environments implicitly co-orchestrates the perceptual experiences of the child.

Such socio-cultural socialization is not limited to early childhood, however, as socialization processes continue during preschool. This is where practices in green areas may be defined both by formal didactics (e.g., Higgins, 2009) and the practices displayed in different families by classmates, as Carlone et al. (2015) shows.

## Cultural Attitudes

However, socio-cultural processes also work on a far larger cultural scale (e.g., Buijs et al., 2009; Kloek et al., 2015). Obviously, in the modern discourse, nature is often articulated alongside concepts such as climate change, sustainability, and the Anthropocene, and in opposition to society, technology, and artificial intelligence (e.g., Steffen et al., 2007; Schilhab, 2015b, 2017b,c). Today, natural environments are considered to offer peace and quiet and especially time off from the stressful rat race that seems to dominate human life (e.g., Pearson and Craig, 2014). The natural environment replaces screen time with bodily activity and therefore ideally counteracts obesity and other welfare diseases (Maller et al., 2006).

Historically, nature has been attributed quite different qualities. In the industrial age, nature as a concept was perceived as a battlefield to be conquered and brought under the control of humanity (Steinberg, 1986; Moore, 2017).

The historical variability in the conception of nature also points to cultural aspects of how we conceive of nature. It is more than likely that in certain countries, both geographical and socio-economic parameters have hugely influenced the qualities attributed to nature (e.g., Skar et al., 2016).

For example, Denmark, where the authors live, is not at risk of largescale earthquakes, volcanic activity, or extreme weather conditions. There are no mountain creeks, avalanches, or underground caves, and only a few actual cliffs. Neither does the fauna contain large predators such as grizzly bears, Bengali tigers, or crocodiles, nor extreme herbivores like hippopotamuses, herds of wildebeests, or swarms of locusts. Along with ectoparasitic ticks, the sea may present the more imposing and dangerous part of nature in Denmark. This said, for a long time, nature has not posed any noteworthy risk to the lives of Danes. In such conditions, we conjecture, the understanding of nature as relaxing and accommodating is especially prone to develop (e.g., “dwelling habitus,” Aner, 2016).

## Concluding Remarks

Summing up, we conjecture that natural environments exert their influence on cognitive states via actual sensory interactions, the socio-cultural perception learned through embodied practices, and the large-scale imaginations held by society and culture. That the comprehension of green spaces is more closely connected with socio-cultural expectations than mere physical qualities has been pointed out convincingly in a recent study from New Zealand, showing how nature may become associated with crime (Fleming et al., 2016).

Obviously, the multiple sources founding the environmental impact on cognitive states do not invalidate the claims of ART. One way to explain the apparent instinctual automaticity often found relating natural environments with particular cognitive states is that socio-cultural factors tend to blend into the tacit knowledge of the individual. As part of the perceptual activity, they front the atmosphere and enrich the conditions for learning in particular interpretations. Eventually, at the level of the individual, these human-based conditions appear innate (Lin et al., 2014).

The question remains as to whether mono-causal relations between the physical environment and cognitive states are ever realized. In other words, can the processing of perceptually available components of the physical environment ever occur in isolation from socio-cultural processes, or is the physical environment undeniably nested within socio-cultural processes through learning (e.g., Lidskog, 1998)? The answer is in need of basic research on the extent to which perceptual processes are modified by learning and whether socio-cultural practices perfuse every part of life.

## Author Contributions

TS and GE conceived of the study. TS and GE contributed conception of the study. TS wrote the first draft of the manuscript. GE wrote sections of the manuscript. Both authors contributed to manuscript revision, read and approved the submitted version.

### Conflict of Interest Statement

The authors declare that the research was conducted in the absence of any commercial or financial relationships that could be construed as a potential conflict of interest.

## Footnotes

^1^Please note, qualifying conditions such as “being away,” “extent,” “fascination,” and “compatibility” must be met for natural environments to facilitate stimulus-dependent attention (Kaplan, 1995). “Being away” accentuates the distancing of oneself from the activities that lead to mental fatigue, whereas “extent” expresses the need of the putatively restorative site to be sufficiently materially “rich” to be perceived as a coherent structure. “Fascination” is the attraction of attention that does not require effort and no inhibition of competing stimuli, while “compatibility” denotes the co-occurrence of what the individual is trying to achieve and the affordances provided by the environment.

^2^Making sense of places is far more nuanced than the picture adopted here. Please refer to Cross (2015) for a more systematic categorisation.
